# Prehospital Lactate Predicts Need for Resuscitative Care in Non-hypotensive Trauma Patients

**DOI:** 10.5811/westjem.2017.10.34674

**Published:** 2018-02-12

**Authors:** Alexander E. St. John, Andrew M. McCoy, Allison G. Moyes, Francis X. Guyette, Eileen M. Bulger, Michael R. Sayre

**Affiliations:** * University of Washington, Division of Emergency Medicine, Seattle, Washington; † University of Pittsburgh, Department of Emergency Medicine, Pittsburgh, Pennsylvania; ‡ University of Washington, Division of Acute Care Surgery, Seattle, Washington

## Abstract

**Introduction:**

The prehospital decision of whether to triage a patient to a trauma center can be difficult. Traditional decision rules are based heavily on vital sign abnormalities, which are insensitive in predicting severe injury. Prehospital lactate (PLac) measurement could better inform the triage decision. PLac’s predictive value has previously been demonstrated in hypotensive trauma patients but not in a broader population of normotensive trauma patients transported by an advanced life support (ALS) unit.

**Methods:**

This was a secondary analysis from a prospective cohort study of all trauma patients transported by ALS units over a 14-month period. We included patients who received intravenous access and were transported to a Level I trauma center. Patients with a prehospital systolic blood pressure ≤ 100 mmHg were excluded. We measured PLac’s ability to predict the need for resuscitative care (RC) and compared it to that of the shock index (SI). The need for RC was defined as either death in the emergency department (ED), disposition to surgical intervention within six hours of ED arrival, or receipt of five units of blood within six hours. We calculated the risk associated with categories of PLac.

**Results:**

Among 314 normotensive trauma patients, the area under the receiver operator characteristic curve for PLac predicting need for RC was 0.716, which did not differ from that for SI (0.631) (p=0.125). PLac ≥ 2.5 mmol/L had a sensitivity of 74.6% and a specificity of 53.4%. The odds ratio for need for RC associated with a 1-mmol/L increase in PLac was 1.29 (95% confidence interval [CI] [0.40 – 4.12]) for PLac < 2.5 mmol/L; 2.27 (1.10 – 4.68) for PLac from 2.5 to 4.0 mmol/L; and 1.26 (1.05 – 1.50) for PLac ≥ 4 mmol/L.

**Conclusion:**

PLac was predictive of need for RC among normotensive trauma patients. It was no more predictive than SI, but it has certain advantages and disadvantages compared to SI and could still be useful. Prospective validation of existing triage decision rules augmented by PLac should be investigated.

## INTRODUCTION

The decision of whether to triage an injured patient to a trauma center can be difficult, and most emergency medical system (EMS) agencies rely on standardized decision-making systems.^1^ Traditional trauma triage systems rely heavily on vital sign abnormalities to identify patients in need of a trauma center. Shock index (SI) is one vital-sign marker that has been identified as an early predictor of severe injury.^2^ However, multiple studies have demonstrated that vital signs are limited in this role.^1^,^3^–^8^

Field measurement of serum lactate concentration could be of potential benefit in accurately identifying patients with more severe injury and need for resuscitative care (RC). Lactic acid is a byproduct of anaerobic metabolism and is a marker of inadequate tissue oxygenation or shock. Technological advancements have led to the development of rapid, portable, lactate assays, permitting lactate measurement in the prehospital and early clinical setting. Previous studies have demonstrated that elevated prehospital and emergency department (ED) lactate levels are predictive of poor outcomes in several populations: septic patients, cardiac arrest patients, and general medical patients.^9^–^15^ Prehospital lactate has also been validated in two populations of patients selected for severe injury: those transported by helicopter and those with prehospital hypotension (systolic blood pressure ≤ 100 mmHg).^16^–^18^

However, the prognostic utility of a prehospital lactate level has not been studied systematically in a population of normotensive trauma patients encountered by ground advanced life support (ALS) crews. Of particular interest is the question of whether the test can risk-stratify normotensive trauma patients and retain specificity for the need for RC when applied to a much broader population with a lower overall prevalence of severe injury than those previously studied. (Note: For the purposes of this paper, the term “normotensive” should be taken to mean any patient with systolic blood pressure > 100 mmHg.) We sought to determine the test characteristics of prehospital lactate levels for predicting need for resuscitative care among a broad population of normotensive trauma patients being transported by ground ALS units.

## METHODS

This study was approved by the University of Washington Institutional Review Board. It was a retrospective analysis of a prospective cohort study of all trauma patients transported by ground ALS units of the Seattle Fire Department between June 24, 2011, and August 21, 2012. In this two-tiered EMS system, ALS treatment and transport to a trauma center is triggered by a significant mechanism of injury, Glasgow Coma Scale ≤ 12, vital-sign abnormalities, neurovascular deficits, and injury pattern, in keeping with Washington State Department of Health Prehospital Trauma Guidelines.^19^ Patients excluded from lactate measurement were those with age less than 15 years; obvious isolated, penetrating head trauma; drowning; asphyxia caused by hanging; burns greater than 20% body surface area; or known prisoner status. We also excluded patients with prehospital systolic blood pressure ≤ 100 mmHg because they had already been analyzed in a prior study with this population showing strong correlation between lactate and outcomes.^17^ This allowed our study to determine the lactate test characteristics among a population that might not be preferentially transported to a high-level trauma center.

Serum lactate levels were drawn upon placement of an intravenous line. A drop of blood was placed on a test strip, which was inserted into a handheld measurement device (Lactate Pro, Arkray Inc., Kyoto, Japan). The Lactate Pro meter is similar in size and operation to the glucometers used by our EMS agencies and has a run time of 60 seconds.^20^ EMS and hospital providers recorded the test result in the patient’s chart but were instructed not to change care based on the number. Data were entered into a local database created specifically for study of prehospital lactate performance. All ALS patients in this catchment area were transported to a single Level I trauma center, Harborview Medical Center. Clinical variables from the time period after ED arrival through death or hospital discharge were obtained by review of electronic health records.

**Population Health Research Capsule**

What do we already know about this issue?

Prehospital triage decisions for trauma patients are based on vital signs, but this misses some injuries. Prehospital lactate measurement could improve this process.

What was the research question?

How predictive is prehospital lactate in predicting severe injury in normotensive trauma patients?

What was the major finding of the study?

Prehospital lactate was predictive of severe injury with reasonable sensitivity and specificity.

How does this improve population health?

This will help inform EMS medical directors of the potential benefits and risks of incorporating prehospital lactate measurement into their trauma triage protocols.

The primary clinical outcome of interest was need for RC. This was defined as death in the ED, disposition to operating room or interventional radiology within six hours, or transfusion of five units of any blood product within six hours of ED arrival. This outcome was used in the prior study of prehospital lactate in hypotensive trauma patients.^17^ It is used here both for consistency to facilitate comparison of results to the prior study and because we believe it accurately defines a population of injured patients that requires high-level trauma care.

In the primary analysis, we defined the normal distribution of prehospital lactate. Subsequently, we evaluated its ability to predict the need for RC and compared it to the same predictive ability of shock index (SI = heart rate [HR] / systolic blood pressure [SBP]) by calculating the area under the curve of the receiver operator characteristic (AUROC) for each. The AUROCs were compared using the DeLong-DeLong-Clarke-Pearson method.^21^ We also calculated the sensitivity and specificity of a prehospital lactate level of 2.5 mmol/L or greater. This cutoff has been previously validated in trauma populations and was chosen to maximize the generalizability of our results.^16^,^22^ The optimal cutoff point for the study population was also calculated by selecting the point visually determined to most closely represent an inflection point in the ROC curve that minimized loss of sensitivity but maximized specificity.

In planned secondary analyses, the AUROCs were calculated and compared in the predefined subpopulations of blunt and penetrating injury patients. We also calculated the AUROCs for prehospital lactate and SI for predicting the alternative outcomes of moderate and severe injury, as defined by injury severity score (ISS) > 9 and 15, respectively. These cutoffs were chosen because they have been used historically to represent moderate and severe injury and have been validated to be useful surrogates for patients who will benefit from triage to a trauma center.^23^–^28^

Finally, we did an exploratory analysis to further investigate the relationship between prehospital lactate and likelihood of need for RC. For this analysis, a multivariate logistic regression was performed that included need for RC as its outcome and a linear spline of lactate with knots at 2.5 and 4 mmol/L as the predictor of interest. We chose this model to allow for a possible non-linear relationship between lactate concentration and risk of need for RC. Cutpoints were previously validated in unrelated studies, indicating that they represent separation points of trauma patients with different outcomes.^29^ We used Wald tests to determine significance.

## RESULTS

**Figure 1:**
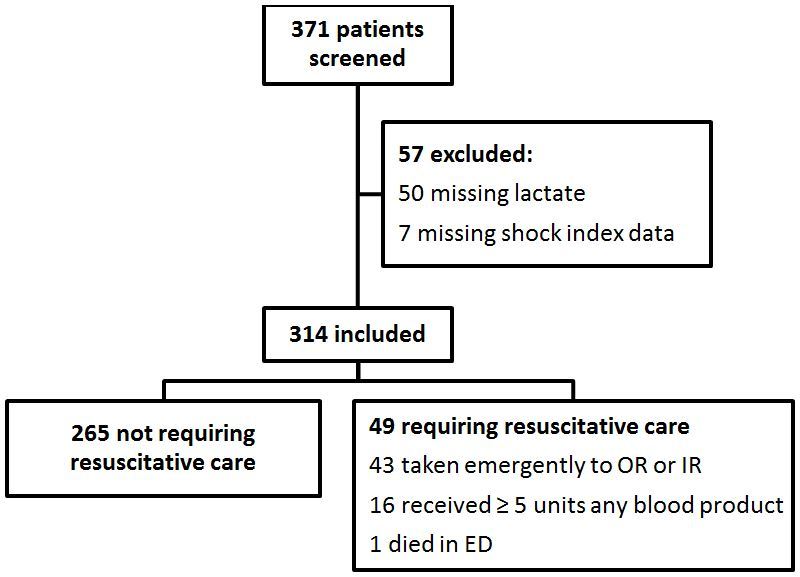
Cohort of patients enrolled in a study of the relationship between prehospital lactate levels and the need for resuscitative care. OR, operating room. IR, interventional radiology. ED, emergency department.

We screened 371 patients for enrollment, and 314 were included in analysis. Of the 57 excluded patients, 50 were excluded for a missing prehospital lactate. The reason given for a missing lactate in those 50 patients was paramedic failure to run the test in 19 patients; ongoing cardiopulmonary resuscitation in 12 patients; unsecure scene in three patients; unstable vital signs in three patients; prolonged extrication in two patients; immediate adjacency to the trauma center in one patien;, and device malfunction in one patient. No reason was provided for eight patients. We also excluded seven patients who were missing a recorded initial HR or SBP necessary to calculate SI. Figure 1 summarizes inclusion/exclusion numbers. Demographic, injury, and hospital data for all included patients are summarized in the table.

**Figure 2:**
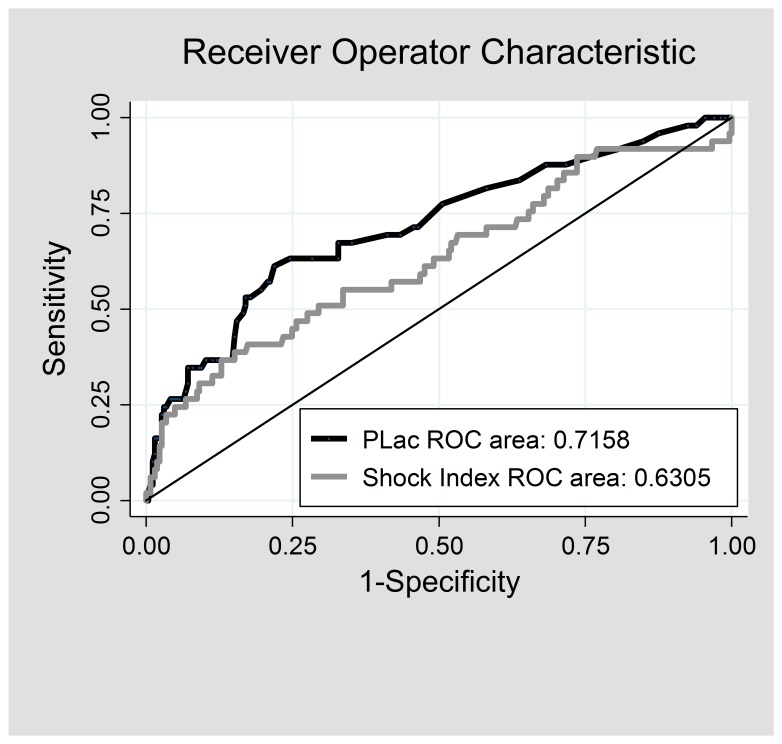
Receiver operator characteristic curves for the prediction of need for resuscitative care by prehospital lactate level and shock index.

The AUROC for prehospital lactate prediction of need for RC was 0.716 (95% confidence interval [CI] [0.632 – 0.800]) and for SI was 0.631 (95% CI [0.537 – 0.724]) (Figure 2). The AUROC for prehospital lactate did not differ from that for SI (*p*=0.125). Among normotensive patients, a prehospital lactate level of 2.5 mmol/L or greater had a sensitivity of 74.6% and specificity of 53.4% for predicting need for RC. Increasing the lactate cutoff level to 3.0, where there is an inflection point in the ROC curve, resulted in improvement in specificity (66.9%) with only modest change in sensitivity (70.9%). In this population, SI of 0.9 or greater (a commonly recognized marker for severe injury in trauma patients) had low sensitivity for predicting need for RC (30.8%) but high specificity (89.9%). Being positive for *either* prehospital lactate level of 2.5 mmol/L or greater *or* SI of 0.9 or greater had only slightly different sensitivity (77.6%) and specificity (49.8%) than with prehospital lactate prediction alone.

Looking at each of the individual outcomes defining need for RC, prehospital lactate had similar predictive power for each. The AUROC for predicting need for emergent surgery was 0.721 (95% CI [0.630 – 0.811]), for predicting transfusion of five units of blood products was 0.785 (95% CI [0.669 – 0.901]) and for predicting death in the ED was 0.863 (95% CI could not be calculated, because there was only one event).

Among the 260 blunt injury patients, the AUROC for prehospital lactate prediction of need for RC was 0.732 (95% CI [0.637 – 0.827]), which was not significantly different from that for SI at 0.657 (95% CI [0.545 – 0.769]) (p=0.121). Among the 54 penetrating injury patients, the AUROC for prehospital lactate was 0.636 (95% CI [0.473 – 0.798]) and for SI was 0.550 (95% CI [0.374 – 0.726]) (p=0.478). In predicting the secondary clinical outcome of moderate injury (ISS > 9), the AUROC for prehospital lactate was 0.592 (95% CI [0.528 – 0.655]) and for SI was 0.594 (95% CI [0.530 – 0.657]) (p=0.954). In predicting severe injury (ISS > 15), the AUROC for prehospital lactate was 0.648 (95% CI [0.580 – 0.716]) and for SI was 0.647 (95% CI [0.576 – 0.718]) (p=0.979).

In the exploratory analysis, the odds ratios for need for RC associated with a 1-mmol/L increase in prehospital lactate concentration was 1.29 (95% CI [0.40 – 4.12], p=0.666) for lactate levels less than 2.5 mmol/L, 2.27 (95% CI [1.10 – 4.68], p=0.027) for lactate levels between 2.5 and 4.0 mmol/L, and 1.26 (95% CI [1.05 – 1.50], p=0.011) for lactate levels greater than 4.0 mmol/L.

## DISCUSSION

In this retrospective cross-sectional analysis of normotensive trauma patients who met criteria for ALS transport, prehospital lactate concentration as measured by a handheld, point-of-care (POC) assay was predictive of the need for RC. Lactate offered no significant performance benefit over shock index by AUROC. However, we propose that it has several advantages over SI. First, it is less prone to error and easier to calculate in real-time during a patient transport. Second, a PLac cutoff of 3.0 offered superior sensitivity (70.9%) over the commonly used SI cutoff of 0.9 (30.8%). Though this came at a modest cost in specificity (66.9 vs. 89.9%), the emphasis for a screening tool in this population is to avoid false-negatives. Third, because prehospital lactate has been demonstrated to be a useful adjunct in the triage of hypotensive ALS transports, its use in normotensive ALS transports could simplify protocols to include a prehospital lactate for any ALS transport. These advantages should be weighed against the disadvantages of cost of equipment and assay, requirement for training, additional time to run the assay, and potential distraction from directly caring for the patient.

The POC meter used in this study cost approximately $300 and required about $2 per use as a disposable cartridge. Our results are similar to those previously seen in hypotensive trauma patients, though each test (prehospital lactate and SI) had slightly lower AUROCs in this non-hypotensive population, likely owing to the rarer need for RC. This is the first prehospital study to examine the ability of prehospital lactate to risk-stratify normotensive trauma patients.

The lactate cutoff of 2.5 mmol/L that has been validated in other trauma populations had a high sensitivity at 75% and maintained specificity at 53%. This indicates that the test may have future value in decreasing undertriage without paying a heavy price in overtriage. One of the critical questions in extending the application of this test to a lower-acuity trauma population than previously studied is whether there would be a major decrease in specificity. Raising the cutoff level to 3.0 mmol/L led to a large increase in specificity with only a small cost paid in sensitivity in this population. However, it is important to note that the same is likely not true in the larger population that includes hypotensive patients. Prospective testing would be needed in the target population with specific comparison of a triage protocol incorporating the prehospital lactate to current triage guidelines.

In the subpopulation secondary analyses, prehospital lactate maintained a high AUROC for both blunt and penetrating patients. Prehospital lactate had no significant difference in performance compared to SI in the blunt or penetrating injury population, though there was a p-value of 0.121 trending toward superior performance in blunt injury. It is difficult to interpret these results in light of the smaller subgroup sample sizes and unknown power due to the lack of prior data in this population, particularly in the penetrating injury group. However, given that most triage criteria direct all penetrating injuries proximal to the knee or elbow to trauma centers already, the test performance comparison in this group might be less relevant.

When examining the prediction of the secondary clinical outcomes of moderate and severe injury (ISS > 9 and 15), prehospital lactate had markedly worse performance. The reasons for this are unclear but could include the fact that hypotensive patients were excluded, making a high ISS more likely representative of extremity and facial injuries less likely to cause shock and limiting the utility of lactate measurement. However, ultimately the high ISS outcome has been used in prior studies as a surrogate measurement predictive of need for RC; in our study, the lactate concentration was directly measured against clinical need for RC, making the primary analysis more impactful than this one.

The exploratory analysis showed that increases in prehospital lactate were associated with increased risk of need for RC only above lactate levels of 2.5 mmol/L. It also showed there is more risk associated with a 1-mmol/L increase in lactate concentration in the range of 2.5 to 4.0 mmol/L, and the increase in risk decreases in the higher lactate ranges. This is both logical, given the relatively high baseline risk associated with any lactate > 4.0, and in keeping with prior findings in a hypotensive trauma population.^17^

In conjunction with previous studies on prehospital lactate in trauma patients, these findings suggest that prehospital lactate could improve overall triage for ALS patients, and we suggest that it should be investigated prospectively as a rapid test in the field to identify occult shock. Among patients meeting local criteria for ALS transport, future investigation should test the integration of prehospital lactate into existing field triage decision rules to determine accuracy in the decision to transport to a trauma center. To account for the possible lack of improvement provided by prehospital lactate testing in the normotensive ALS transport population, future trials should include a planned subgroup analysis of normotensive patients. If found to be beneficial, testing could also be investigated for use by basic life support (BLS) providers to identify patients with occult shock requiring ALS transport, given that the tasks required for testing are within the BLS skillset. The role of the test should be further investigated primarily to prevent undertriage, as its test characteristics show that this would likely come at a relatively low cost of overtriage, even among normotensive trauma patients. A positive result could also hold promise for triggering more aggressive field treatment, including the earlier use of prehospital blood products in EMS systems with the capability to do so, though this would also require further study.

## LIMITATIONS

This study has several limitations. First, although the data were collected prospectively, this is a retrospective analysis, which limits some of the granularity of the data. Second, because we lacked the ability to determine specific operative procedures being performed, we used disposition to OR within six hours as a surrogate for surgical hemorrhage control. This would inadvertently include the uncommon patient undergoing a non-emergent surgical procedure within six hours of ED arrival, such as open reduction internal fixation, but that would only bias the results towards a worse sensitivity for prehospital lactate. Third, the definitions for the composite outcome of need for RC were chosen subjectively. They were chosen both for ease of comparison to prior literature and because the authors felt they accurately represented a population requiring higher-level trauma care, but it could be argued that this is not an optimally targeted population.

Fourth, this study still only included patients meeting ALS criteria in a two-tiered system. It is important to note that, although this population includes normotensive trauma patients, the findings could not be extended to the population transported by BLS in our system. Furthermore, the impact of inclusion of prehospital lactate into any triage protocol would need to be investigated prospectively before it could be recommended for implementation. Fifth, while the lactate meter used in the study is Clinical Laboratory Improvement Amendment-waived, it is no longer commercially available. The Lactate Pro is operated identically to the POC glucose meter used on ambulances with minimal operational error. The devices are robust and reliable with a low failure rate.^20^ During the study period, the meter did not require calibration. Future lactate meters available for prehospital use may be more complicated both in their operation and their administrative overhead.

Finally, we did not have access to all prehospital data, so we were unable to compare the performance of the current triage algorithm to what the performance would have been with a triage algorithm incorporating the prehospital lactate data. This study also has several important strengths, including prospective collection of the data and the single receiving center for patients, enhancing the collection of detailed in-hospital data.

## CONCLUSION

In conclusion, this study suggests that prehospital lactate could be useful in risk-stratifying normotensive trauma patients. Prospective validation of existing triage decision rules augmented by prehospital lactate should be a focus of future investigation.

## Figures and Tables

**Table:** Demographic, injury, and hospital data of included patients

Characteristic	Study population (n=314)
Age, years, median (IQR)	35.5 (25–51)
Male, n (%)	228 (72.6)
Race, n (%):	
White	181 (57.6)
Black	47 (15.0)
Hispanic	20 (6.4)
Asian	13 (4.1)
Pacific Islander	11 (3.5)
Native American	5 (1.6)
Other	1 (0.3)
Unknown	36 (11.5)
Mechanism of injury, n (%):	
Blunt	260 (82.8)
Fall	68 (21.7)
Motor vehicle collision	66 (21.0)
Pedestrian struck	38 (12.1)
Assault	28 (8.9)
Bicycle collision	23 (7.3)
Motorcycle collision	19 (6.1)
Other blunt injury	25 (8.0)
Penetrating	54 (17.2)
Gunshot wound	29 (9.2)
Stab wound	21 (6.7)
Other penetrating injury	4 (1.3)
Injury severity score, median (IQR)	9 (5–19)
Initial emergency department laboratory values:*	
Hematocrit, median (IQR)	40 (37–43)
International normalized ratio, median (IQR)	1.0 (1.0–1.1)
pH, median (IQR)	7.35 (7.30–7.41)
Hospital lactate concentration, mmol/L, median (IQR)	3.0 (2.2–4.7)
Emergency department care:	
Crystalloid volume infused in first 6 hours, mL, median (IQR)	1,500 (1,000–2,100)
Received pRBC transfusion in first 6 hours, n (%)	30 (9.6)
Outcomes	
Emergency department length of stay, minutes, median (IQR)	258 (184–391)
Death in emergency department, n (%)	1 (0.3)
Hospital length of stay, hours, median (IQR)	44.1 (7.1–155.5)
Intensive care unit days, median (IQR)	0 (0–2)
In-hospital mortality, n (%)	15 (4.8)
Hospital discharge location if alive, n (%):	
Home / self-care	236 (75.2)
Skilled nursing facility	26 (8.3)
Inpatient rehabilitation center	11 (3.5)
Other	12 (3.8)
Not documented	29 (9.2)

*IQR*, interquartile ratio, *pRBC,* packed red blood cells.

* Many laboratory values were not run in all patients, resulting in absence of reported values ranging from <1% (hematocrit) to 56% (hospital lactate concentration).
